# Directed Self-Assembly of Trimeric DNA-Bindingchiral Miniprotein Helicates

**DOI:** 10.3389/fchem.2018.00520

**Published:** 2018-10-30

**Authors:** Jacobo Gómez-González, Diego G. Peña, Ghofrane Barka, Giuseppe Sciortino, Jean-Didier Maréchal, Miguel Vázquez López, M. Eugenio Vázquez

**Affiliations:** ^1^Centro Singular de Investigación en Química Biolóxica e Materiais Moleculares (CiQUS), Departamento de Química Inorgánica, Universidade de Santiago de Compostela, Santiago de Compostela, Spain; ^2^Centro Singular de Investigación en Química Biolóxica e Materiais Moleculares (CiQUS), Departamento de Química Orgánica, Universidade de Santiago de Compostela, Santiago de Compostela, Spain; ^3^Departament de Química, Universitat Autònoma de Barcelona, Cerdanyola, Spain; ^4^Dipartimento di Chimica e Farmacia, Università di Sassari, Sassari, Italy

**Keywords:** metallopeptide, self-assembly water, DNA recognition, enantioselectivity, peptide motifs, coordination chemistry

## Abstract

We propose that peptides are highly versatile platforms for the precise design of supramolecular metal architectures, and particularly, for the controlled assembly of helicates. In this context, we show that the bacteriophage T4 Fibritin foldon (T4Ff) can been engineered on its N-terminus with metal-chelating 2,2′-bipyridine units that stereoselectively assemble in the presence of Fe(II) into parallel, three-stranded peptide helicates with preferred helical orientation. Modeling studies support the proposed self-assembly and the stability of the final helicate. Furthermore, we show that these designed mini-metalloproteins selectively recognize three-way DNA junctions over double-stranded DNA.

## Introduction

Peptides are excellent supramolecular building blocks that encode precise structural and functional information within their amino acid sequence. Accordingly, researchers have explored diverse peptide motifs, such as coiled-coils, β-hairpins, or peptide amphiphiles, as the basis of biofunctional devices and materials (Matsuura et al., 2005, 2010; Gazit, 2007; Ulijn and Smith, 2008; Apostolovic et al., 2010; Robson Marsden and Kros, 2010; Boyle and Woolfson, 2011; Lai et al., 2012; Pazos et al., 2016). Curiously, despite the enormous potential for controlling stereochemistry, nuclearity and stoichiometry, the controlled supramolecular assembly of inorganic complexes with peptide motifs has been somewhat overlooked, and only a handful of systems based on modified coiled-coil motifs have been reported (Lieberman and Sasaki, 1991; Ghadiri et al., 1992; Li et al., 2000; Peacock et al., 2012; Ball, 2013; Berwick et al., 2014; Luo et al., 2016). On the other hand, helicates are discrete metal complexes in which one or more organic ligands are coiled around—and coordinating—two or more metal ions (Piguet et al., 1997; Albrecht, 2001, 2005) as a result of ligand coiling, helicates are inherently chiral species that can appear as two enantiomers according to the orientation in which the ligands twist around the helical axis defined by the metal centers. Besides their intrinsic interest in basic supramolecular chemistry, helicates have shown promising DNA-binding properties that have been associated with antimicrobial and antitumoral effects (Howson et al., 2012; Kaner et al., 2015). However, more than 20 years after the pioneering studies by Prof. Jean-Marie Lehn (Lehn et al., 1987; Ulijn and Smith, 2008), helicates are still not viable alternatives to traditional DNA-binding agents. The slow development in the applied chemistry of metal helicates ultimately derives from the shortcomings associated with the classic synthetic approaches with organic ligands that complicate the structural control of the final helicates (i.e., oligomerization state, relative orientation of asymmetric ligands, supramolecular helicity) and hampers their efficient structural and functional optimization. Indeed, despite some noteworthy examples (Haino et al., 2009; Cardo et al., 2011; Howson et al., 2012; Chen et al., 2017; Mitchell et al., 2017; Guan et al., 2018), no general approach for the efficient and versatile stereoselective synthesis of helicates is yet available, making of these systems a challenging test case to demonstrate the potential of peptides for the controlled assembly of metallostructures.

Our strategy relied in the selection of a synthetically-accessible and structurally well-defined trimeric peptide domain as scaffold for the programmed assembly of the helicate. As an alternative (and orthogonal) platform to the ubiquitous leucine zippers, we focused our attention on the C-terminal domain of the bacteriophage T4 Fibritin foldon (T4Ff), a trimeric β-propeller-like structure formed by the self-assembly of a short 27-amino acid peptide (Tao et al., 1997; Papanikolopoulou et al., 2004; Habazettl et al., 2009). The intrinsic stability and structural resilience of the T4Ff scaffold has been exploited for the stabilization of trimeric structures of a number of peptides and engineered proteins (Stetefeld et al., 2003; Du et al., 2011; Berthelmann et al., 2014; Kobayashi et al., 2015), and given those precedents we envisioned that the T4Ff could also be used as a robust platform for the programmed assembly of chiral dinuclear helicates, thus offering an alternative for the integration of coordination and peptide chemistry beyond other widely explored peptide scaffolds.

## Materials and methods

### General

All reagents were acquired from the regular chemical suppliers. All solvents were dry and synthesis grade, unless specifically noted (NH_4_)Fe_2_(SO_4_)_2_ • 6 H_2_O salt from *Sigma-Aldrich* was used as Fe(II) ion source. Reactions were followed by analytical UHPLC-MS with an *Agilent 1200* series LC/MS using a *SB C18* (1.8 μm, 2.1 × 50 mm) analytical column from *Phenomenex*. Standard conditions for analytical UHPLC consisted on a linear gradient from 5 to 95% of solvent B for 12 min at a flow rate of 0.35 mL/min (A: water with 0.1% TFA, B: acetonitrile with 0.1% TFA). Compounds were detected by UV absorption at 222, 270, and 330 nm. Electrospray Ionization Mass Spectrometry (ESI/MS) was performed with an *Agilent 6120 Quadrupole* LC/MS model in positive scan mode using direct injection of the purified peptide solution into the MS detector.

### Computational methods

The model for the ΛΛ-[**(**β**AlaBpy)**_2_**-T4Ff**]_3_Fe${}_{2}^{+4}$ helicate was built with UCSF chimera1.12 (Pettersen et al., 2004), starting from the NMR resolved structure of the trimeric Foldon of the T4 phagehead fibritin (PDB code: 1RFO) mutating the carboxyl C-Termini to amide groups (see *Results and Discussion* section). Based on previous work, the model of ΛΛ-[**(**β**AlaBpy)**_2_**-T4Ff**]_3_Fe${}_{2}^{+4}$ helicate were built connecting the N-termini of the T4Ff peptides. Molecular Dynamics (MD) simulations were set up with the *xleap*, solvating the model with a box of pre-equilibrated TIP3P water molecules and the total charge was balanced with Cl^−^ ions (*ions94.lib* library). The AMBER14SB force field was used for standard residues (Hornak, Abel, Okur, Strockbine, Roitberg and Simmerling., 2006), while the GAFF force field was adopted for the remaining atoms. Fe-bonding force constants and equilibrium parameters were obtained through the Seminario method, using Gaussian09 to compute the geometry and harmonic frequencies at DFT level (Frisch et al., 2010), with the B3LYP functional (Yanai et al., 2004), combined with scalar-relativistic Stuttgart–Dresden SDD pseudopotential and its associated double-ζ basis plus a set of *f* polarization functions for the metal ion (Ehlers et al., 1993). The 6-31G(d,p) basis set was used for H, C, O, and N. Point charges were derived using the RESP (Restrained ElectroStatic Potential) model (Bayly et al., 1993). The force field building operations were carried out using the MCPB.py (Li and Merz, 2016). The solvent and the whole system were sequentially submitted to 3,000 energy minimization steps to relax possible steric clashes. Then, thermalization of water molecules and side chains was achieved by increasing the temperature from 100 K up to 300 K. MD simulations under periodic boundary conditions were carried out during 100 ns with OpenMM engine through OMMProtocol (Eastman et al., 2017; Pedregal et al., 2018). Analysis of the trajectories was carried out by means of *cpptraj* implemented in ambertools16 (Case et al., 2016).

### Solid-phase peptide synthesis (SPPS)

All peptide synthesis reagents, as well as the Fmoc amino acid derivatives were purchased from *GL Biochem* (Shanghai) Ltd., Fmoc-β-Ala-OH was from *Sigma Aldrich*. C-terminal amide natural T4Ff peptides were synthesized following standard Fmoc-peptide synthesis protocols on a 0.1 mmol scale using a 0.5 mmol/g loading *H*-*Rink amide ChemMatrix* resin (35–100 mesh) from *Sigma Aldrich* with a *Liberty Lite* automatic microwave assisted peptide synthesizer from *CEM Corporation*. The amino acids were coupled in 5-fold excess using oxyme as an activating agent. Couplings were conducted for 4 min at 90°C. Deprotection of the temporal Fmoc protecting group was performed by treating the resin with 20% piperidine in DMF for 1 min at 75°C. Once the synthesis is finished, the peptide was acetylated with a solution of 0.8 ml AcOH, 2 ml of DIEA/DMF (0.2 M) and 3.2 ml of DMF. The last non-natural Fmoc-β-Ala-Bpy-OH residues were coupled by hand in 4-fold excess using HATU as activating agent. Each amino acid was activated for 1 min in DIEA/DMF 0.2 M before being added onto the resin. These manual couplings were conducted for 60 min. Deprotection of the temporal Fmoc protecting group was performed by treating the resin with 20% piperidine in DMF for 20 min. Cleavage and deprotection of the peptide were simultaneously performed using standard conditions by incubating the resin for 2.5 h with an acidic mixture containing 50 μL CH_2_Cl_2_, 25 μL of H_2_O, 25 μL of TIS (triisopropylsilane), and 900 TFA μL. The resin was filtered, and the TFA filtrate was concentrated under a nitrogen stream to an approximate volume of 1 mL, and then added onto ice-cold diethyl ether (20 mL). After 10–30 min, the precipitate was centrifuged and washed again with 5 mL of ice-cold ether. The solid residue was dried under argon and redissolved in acetonitrile/water 1:1 (2–5 mL) and purified by semi-preparative RP-HPLC.

Peptides were purified by preparative RP-HPLC with an *Waters 1500* series Liquid Chromatograph using a *Sunfire Prep C18 OBD* (5 μm, 19 × 150 mm) reverse-phase column from *Waters*. Standard conditions for analytical and preparative RP- HPLC consisted on an isocratic regime during the first 2 min, followed by a linear gradient from 15 to 75% of solvent B for 30 min (A: water 0.1% TFA, B: acetonitrile 0.1% TFA). Compounds were detected by UV absorption (222 nm) and by ESI/MS. The fractions containing the products were freeze-dried and their identity was confirmed by ESI/MS and MALDI-TOF. Matrix-assisted laser desorption/ionization mass spectrometry (MALDI/MS) was performed with a Bruker *Autoflex MALDI/TOF* model in positive scan mode by direct irradiation of the matrix-absorbed peptide.

### Spectroscopic measurements

UV measurements were made in a *Jasco V-630* spectrophotometer coupled to a *Jasco ETC-717* temperature controller, using a standard *Hellma* semi-micro cuvette (108.002-QS) with a light path of 10 mm. Measurements were made at 20°C. Luminescence experiments were made with a *Varian Cary Eclipse* Fluorescence Spectophotometer coupled to a *Cary Single Cell peltier accessory (Agilent Technologies)* temperature controller. All measurements were made with a *Hellma* semi-micro cuvette (108F-QS) at 20°C. Circular dichroism measurements were made with a *Jasco J-715* coupled to a *Neslab RTE-111* termostated water bath, using a *Hellma* 100-QS cuvette (2 mm light pass).

### Electrophoretic mobility shift assays

EMSA were performed with a *BioRad Mini Protean* gel system, powered by an electrophoresis power supplies *PowerPac* Basic model, maximum power 150 V, frequency 50–60 Hz at 140 V (constant V). Binding reactions were performed over 30 min in 1.8 mM Tris-HCl (pH 7.5), 90 mM KCl, 1.8 mM MgCl_2_, 0.2 mM TCEP, 9% glycerol, 0.11 mg/mL BSA, and 2.2% NP-40. For the experiments we used 200 nM of the DNAs (twDNA and dsDNA), and a total incubation volume of 20 μL. After incubation for 30 min at room temperature, products were resolved by PAGE using a 10% non-denaturing polyacrylamide gel and 1 × TBE buffer (0.445 M Tris, 0.445 M Boric acid) for 35 min at 25°C, and analyzed by staining with SyBrGold (Molecular Probes: 5 μL in 50 mL of 0.5 × TBE) for 10 min and visualized by fluorescence (*BioRad GelDoc XR*+ molecular imager).

## Results and discussion

As metal-chelating unit we chose 2,2′-bipyridine, a ligand that has been extensively used in coordination chemistry and yields stable complexes with a variety of metal ions (Kaes et al., 2000). Furthermore, we have previously described an Fmoc-protected 2,2′-bipyridine dipeptide derivative that can be readily implemented into standard Fmoc solid-phase peptide synthesis (SPPS) protocols, and have showed that the structure of this chelating unit, in which the 2,2′-bipyridine ligand is integrated in the peptide backbone, effectively couples the conformational preferences of the peptide chain with the geometry of the resulting metal complexes (Rama et al., 2012; Gamba et al., 2013, 2014, 2016; Salvadó et al., 2016).

The chelating 2,2′-bipyridine residue was obtained following an optimized synthetic route (Rama et al., 2012), based on the work carried out by the Newkome and Imperiali groups (Newkome et al., 1997; Torrado et al., 1998). The key step in the synthesis being the desymmetrization of a diethyl [1,1′-biphenyl]-4,4′-dicarboxylate intermediate with hydrazine monohydrate under conditions that allow the selective precipitation of the monocarbohydrazide, which is oxidized into the corresponding azyl azide, and then transformed into a carbamate through a Curtius rearrangement (Rama et al., 2012). Simultaneous hydrolysis of the carbamate and the ester group gives the desired bipyridine amino acid, which is derivatized in the form of a dipeptide to obtain the Fmoc-βAlaBpy-OH building block for increased solubility, stability, and solubility that allow its use following standard solid-phase peptide synthesis protocols (Ishida et al., 2006).

**Figure 1:**
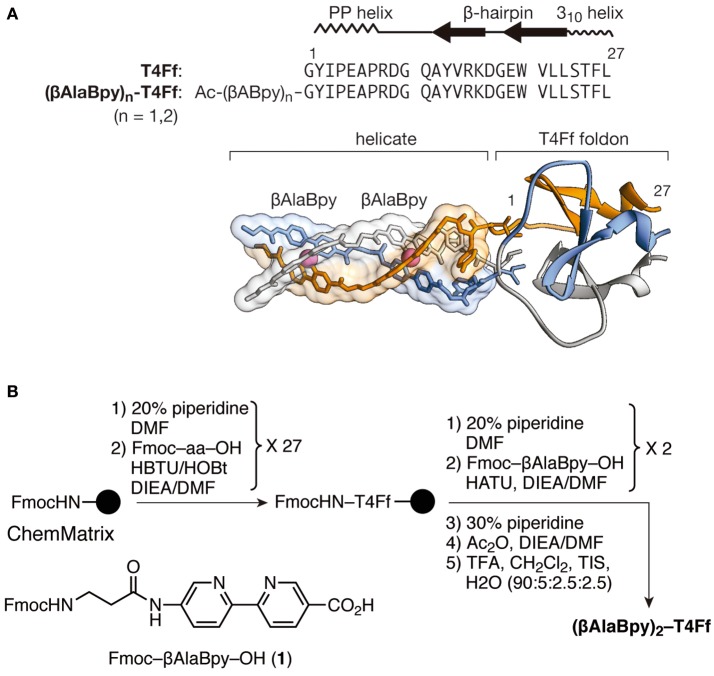
**(A)** Structural elements and sequence of the natural T4Ff, and proposed structure of the **(**β**AlaBpy)**_2_**-T4Ff** helicate at the N-terminus of the T4Ff scaffold. The three chains of the T4Ff are shown with different colors (orange, blue, light gray) for clarity. The ΛΛ- chirality is induced by the natural twisting of the T4Ff N-terminal polyproline helices. **(B)** Synthetic procedure for obtaining the T4Ff helicates, and structure of the chelating Fmoc-βAlaBpy-OH amino acid.

Inspection of the structure of T4Ff (PDB IDs 4NCU or 1RFO; Güthe et al., 2004; Berthelmann et al., 2014) showed that the N-terminal Gly residues are relatively close to each other and could accommodate the chelating 2,2′-bipyridine units without noticeable distortion of the T4Ff scaffold upon metal coordination. Moreover, we envisioned that the natural twist of the N-terminal polyproline helices in the folded T4Ff trimer should induce a ΛΛ-configuration (*M* helicity) on its derived helicate (Tao et al., 1997), which would be the preferred chirality for the efficient recognition of three-way DNA junctions (Oleksy et al., 2006; Gamba et al., 2016). Therefore, we synthesized the desired **(**β**AlaBpy)**_2_**-T4Ff** helicate precursor ligand following standard Fmoc SPPS methods as outlined in Figure 1 (Coin et al., 2007). The final peptide ligand was purified by HPLC and its identity confirmed by ESI-MS.

**Figure 2:**
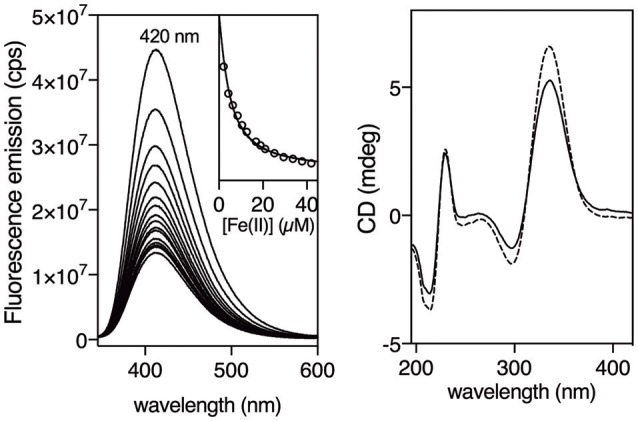
Fluorescence titration of a 3 μM (9 μM monomer) solution of [**(**β**AlaBpy)**_2_**-T4Ff**]_3_ with increasing concentrations of Fe(II). Inset shows emission at 420 nm upon excitation at 305 nm with increasing concentrations of Fe(II), and the best fit to a 1:2 binding mode (Hellman and Fried, 2007; Peberdy et al., 2007). Experiments were made in triplicate. Right. Circular Dichroism of a 6 μM solution (18 μM monomer) of [**(**β**AlaBpy)**_2_**-T4Ff**]_3_ (dashed line) and in the presence of 90 μM Fe(II) (solid line). All experiments were made in 1 mM phosphate buffer, pH 6.5, 10 mM NaCl at 20°C.

Having at hand the desired peptides we proceeded with the study of their metal binding properties. Surprisingly, while 2,2′-bipyridine is weakly emissive, and is even considered non-fluorescent (Dhanya and Bhattacharyya, 1992; Yagi et al., 1994), we found that the asymmetric 5′-amido-[2,2′-bipyridine]-5-carboxamide unit within the βAlaBpy residue was highly emissive, displaying intense band at c.a. 420 nm with a quantum yield of 0.37 (Dong et al., 2017). Additionally, the emission was quenched by coordination to Fe(II) ions, which could be exploited to monitor the formation of the β-annulus helicate. Thus, we recorded the emission spectra of a 3 μM solution (9 μM monomer) of [**(**β**AlaBpy)**_2_**-T4Ff**]_3_ in phosphate buffer (1 mM, pH 6.5) in the presence of increasing concentrations of (NH_4_)_2_Fe(SO_4_)_2_ • 6 H_2_O (Mohr salt) as source of Fe(II) ions (λexc = 305 nm), and observed a concentration-dependent quenching of the emission intensity of the bipyridine ligands. The emission intensity profile of the titration nm could be fitted to a 1:2 binding mode with dissociation constants for the first, and second iron coordination of *K*_*D*1_ = 5.5 ± 3.3 μM and a *K*_*D*2_ = 6.6 ± 0.7 μM, respectively (Figure 2, left; Kuzmic, 1996, 2009). UV/Vis titrations were also qualitatively consistent with the fluorescence data, showing a weak MLCT at about 535 nm in the presence of Fe(II) ions (See Supplementary Material). The formation of the expected [[**(**β**AlaBpy)**_2_**-T4Ff**]_3_Fe_2_]^4+^ complex was also confirmed by mass spectrometry of the final solution of the titrations, which showed a peak at the expected mass of the molecular ion (m/*z* = 11084.6).

In order to study the chirality induction around the metal centers we measured the circular dichroism spectra of the trimeric [**(**β**AlaBpy)**_2_**-T4Ff**]_3_ ligand, and its Fe(II) complex [**(**β**AlaBpy)**_2_**-T4Ff**]_3_Fe${}_{2}^{+4}$. As expected from the original structural analysis, the observed positive Cotton effect at c.a. 330 nm is consistent with the formation of a ΛΛ-helicate. Furthermore, the small change in the CD spectra upon addition of Fe(II) also suggests that the bipyridine ligands are strongly preorganized, even in absence of the metal, and that only a small rearrangement of the chromophores takes place upon coordination (Figure 2, right). This is consistent with earlier computational studies with related bis-bipyridyl peptide ligands, which showed that the bipyridine residues have a large tendency to stack on top of each other (Rama et al., 2012). This stacking interaction will presumably rigidify the bis-bipyridyl trimer and facilitate the helical induction by the foldon domain.

In order to gain some insight into the structure and stability of the peptide helicate we performed Molecular Dynamics (MD) simulations in explicit solvent and periodic boundary conditions (see Methods section for details). The structure of the ΛΛ-[**(**β**AlaBpy)**_2_**-T4Ff**]_3_Fe${}_{2}^{+4}$ unit appears highly stable along all the MD trajectory retaining its helicity conformation and the Fe(II) octahedral coordination geometry. Moreover, the T4Ff scaffold appears stable during the simulation showing no appreciable deformations as a result of the introduction of the artifical (βAlaBpy)_2_ unit. The root-mean square deviation (RMSD) of the whole system was computed along the MD using the minimized initial structures as a reference, the trajectories attain relative stable RMSD after the first ~20 ns, that reach up to 1.99 ± 0.62 Å in average (See Supplementary Material). A cluster analysis was performed on the full length MD experiments showing a predominant conformations occupying about ~40% of the total conformation repartition. Overall, the results highlight that the computed model is very stable along the 100 *ns* of the MD and results consistent with the experimental data. Interestingly, the MD analysis revealed a hinge region with increased flexibility connecting the more rigid helicate and foldon domains, which suggests the replacement of the N-terminal Gly reside for a more conformationally restricted residue in future designs.

**Figure 3:**
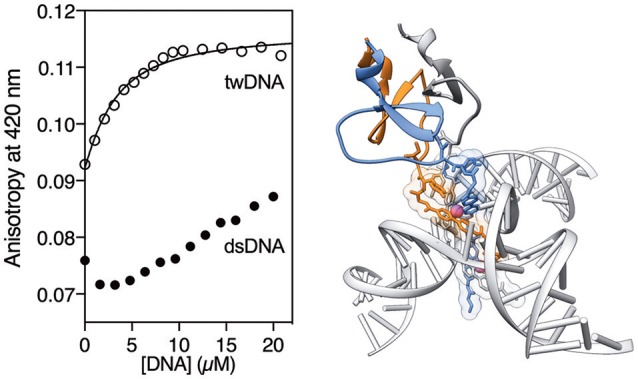
**First** cluster representative frame of the MD trajectory for the ΛΛ-[**(**β**AlaBpy)**_2_**-T4Ff**]_3_Fe${}_{2}^{+4}$ system showing the stable structure of the T4Ff domain. Note the flexible hinge region between the rigid helicate and the T4Ff domain.

**Figure 4:**
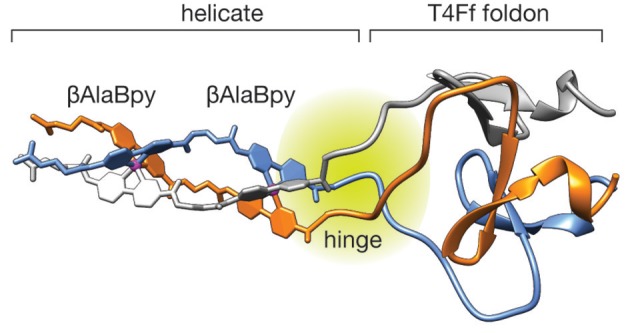
(Left) Anisotropy titration of [**(**β**AlaBpy)**_2_**-T4Ff**]_3_Fe_2_ in 1 mM phosphate buffer, 10 mM NaCl with increasing concentrations of **tw-DNA**. The best fit to a 1:1 binding mode is shown (curve fitting was performed using *DynaFit*).(Kuzmic, 1996, 2009) **tw-DNA** sequences: 5′–CAC CGC TCT GGT CCT C−3′; 5′–CAG GCT GTG AGC GGT G−3′; 5′–GAG GAC CAA CAG CCT G−3′. Right: Model of the interaction between the [**(**β**AlaBpy)**_2_**-T4Ff**]_3_Fe_2_ and the three-way junction, based on the reported pdb structures of an helicate bound to a three-way junction (pdb code 4NCU), and the structure of the fibritin foldon (pdb code 2ET0; Oleksy et al., 2006).

Having made a preliminary characterization of the T4Ff helicate, we studied its DNA binding properties by titrating a 2 μM solution of [**(**β**AlaBpy)**_2_**-T4Ff**]_3_ (6 μM mononer) in the presence of saturating concentrations of Fe(II) according to the previous fluorescence titrations (20 μM) with increasing concentrations of a three-way DNA junction (**tw-DNA**), and measuring the fluorescence anisotropy of the bipyridine fluorophores at 420 nm after each addition of DNA. The titration profile could be fitted to a 1:1 binding mode, with a dissociation constant of 2.17 ± 0.45 μM of the [**(**β**AlaBpy)**_2_**-T4Ff**]_3_Fe_2_ complex to **tw-DNA**. Titrations under the same conditions with a model double stranded DNA (**ds-DNA**) led to a small, monotonic increase in the anisotropy, which is in tune with the the formation of weak complexes or non-specific binding (Figure 3). The low affinity to dsDNA is consistent with previous studies with other helicates (Figure 4; Tuma et al., 1999; Oleksy et al., 2006; Gamba et al., 2016). Control titrations adding with [**(**β**AlaBpy)**_2_**-T4Ff**]_3_ foldon in absence of metal did not show any response to added DNA (See Supplementary Material), thus confirming that the formation of the helicate structure is required for DNA recognition, and the foldon only have a structural role in the formation of the helicate.

**Figure 5:**
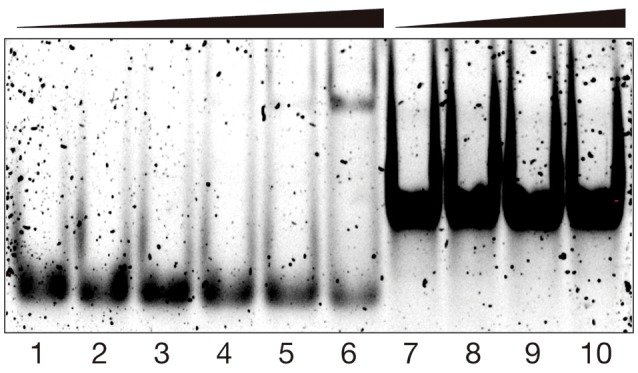
EMSA DNA binding studies results for [**(**β**AlaBpy)**_2_**-T4Ff**]_3_Fe_2_ helicate. Lanes 1–6, 200 nM **tw-Rho-DNA** with 0, 150, 250, 500, 1,000, and 2,000 nM of [**(**β**AlaBpy)**_2_**-T4Ff**]_3_ and 14 eq. of (NH4)_2_Fe(SO_4_)_2_ ∙ 6 H_2_O in each lane; lanes 7–10, 200 nM dsDNA with 0, 500, 1,000, and 2,000 nM of [**(**β**AlaBpy)**_2_**-T4Ff**]_3_ and 14 eq. of (NH4)_2_Fe(SO_4_)_2_ ∙ 6 H_2_O in each lane. Samples were resolved on a 10% nondenaturing polyacrylamide gel and 1 × TBE buffer over 35 min at 25°C, and stained with SyBrGold (5 μL in 50 mL of 0.5 × TBE) for 10 min, followed by fluorescence visualization. Oligonucleotide sequences: **tw-DNA**, 5′–CAC CGC TCT GGT CCT C−3′; 5′–CAG GCT GTG AGC GGT G−3′; 5′–GAG GAC CAA CAG CCT G−3′; **dsDNA** (only one strand shown) 5′–AAC ACA TGC AGG ACG GCG CTT−3′.

In addition to the spectroscopic studies, we also studied the DNA binding properties of the [**(**β**AlaBpy)**_2_**-T4Ff**]_3_Fe_2_ helicate by electrophoretic mobility assays (EMSA) in polyacrylamide gel under non-denaturing conditions (Liebler and Diederichsen, 2004), visualizing the DNA in the gel using *SybrGold* staining (Vázquez et al., 2007). In agreement with the fluorescence titration studies discussed previously, incubation of the target **tw-DNA** with the [**(**β**AlaBpy)**_2_**-T4Ff**]_3_Fe_2_ helicate resulted in the concentration-dependent appearance of a new retarded band, which is consistent with the formation of the expected **tw-DNA**/[**(**β**AlaBpy)**_2_**-T4Ff**]_3_Fe_2_ complex (Figure 5, lanes 1–6). Additionally, the overall intensity of the lanes of the gel is progressively reduced in the presence of increasing concentrations of the [**(**β**AlaBpy)**_2_**-T4Ff**]_3_Fe_2_ complex, which suggests the formation of higher-order aggregates with the three-way junction DNA in the gel conditions (Chanvorachote et al., 2009; Thordarson, 2010). On the other hand, incubation of a model double-stranded DNA with the peptide helicate did not show any new slow-migrating bands (Figure 5, lanes 7-10), which is in agreement with the expected low affinity for this form of DNA, and demonstrates that the small increase observed in the fluorescence anisotropy titration of dsDNA (Figure 4) arises from weak interactions that are not seen at the lower concentrations used in the EMSA experiment.

## Conclusions

In summary, we have shown the potential of small protein domains for the precise structural organization of coordination complexes. Modification of the T4 Fibritin foldon with metal-chelating bipyridines results allows the assembly of unique three-strand helicates in which the parallel orientation of the three helicate ligands is directed by the self-assembled T4Ff domain, and the chirality of the dinuclear helicate (*M* helicity or ΛΛ-configuration in the metal complexes) is selected by the relative orientation of the natural polyproline helices at the N-terminus of the T4Ff trimer. The final supramolecular peptide helicate [**(**β**AlaBpy)**_2_**-T4Ff**]_3_Fe_2_ displays good *in vitro* DNA binding and selectivity toward three-way DNA junctions. We are currently exploring alternative peptide sequences to improve the solubility of the peptide/DNA complexes, and modifications with positively charged residues that might increase the overall affinity.

## Author contributions

JG-G and DGP performed the experimental work (synthesis of the bipyridine building block, peptide synthesis, metal and DNA binding studies), GB did preliminary studies with the (βAlaBpy)2-T4Ff peptide. GS and J-DM did the computational work and contributed to the preparation of the final manuscript. MVL and MEV conceived the project, supervised the experimental work. MEV wrote the manuscript with the collaboration of MVL, and prepared the graphic material.

### Conflict of interest statement

The authors declare that the research was conducted in the absence of any commercial or financial relationships that could be construed as a potential conflict of interest.
