# Supplementary material for: Directed Self-Assembly of Trimeric DNA-Bindingchiral Miniprotein Helicates
Source: Front Chem. 2018 Oct 30;6:520. doi: 10.3389/fchem.2018.00520 (PMC6218460; doi:10.3389/fchem.2018.00520)
Supplement: Supplementary file 1 [file Data_Sheet_1.pdf]

# Supporting Information

# Directed self-assembly of trimeric DNA-binding chiral miniprotein helicates

*Jacobo Gómez-González,[1] Diego G. Peña,[2] Ghofrane Barka,[1] Giuseppe Sciortino,[3,4] Jean-Didier Maréchal,[3,*] Miguel Vázquez López,[1,*] M. Eugenio Vázquez,[2,*]*

1 Centro Singular de Investigación en Química Biolóxica e Materiais Moleculares (CiQUS), Departamento de Química Inorgánica, Universidade de Santiago de Compostela. 15782 Santiago de Compostela, Spain.

2 Centro Singular de Investigación en Química Biolóxica e Materiais Moleculares (CiQUS), Departamento de Química Orgánica, Universidade de Santiago de Compostela. 15782 Santiago de Compostela, Spain.

3 Departament de Química, Universitat Autònoma de Barcelona, 08193 Cerdanyola, Spain.

4 Dipartimento di Chimica e Farmacia, Università di Sassari, Via Vienna 2, I-07100 Sassari, Italy.

# Peptide synthesis

**(βAlaBpy)$_2$-T4Ff**: Ac-(βAlaBpy)$_2$-GYIPEAPRDGQAYVRKDGEWVLLSTFL-NH$_2$

**(βAlaBpy)$_2$-T4Ff**: ESI-MS (m/z) $[M+H]^+$ calcd. for $C_{172}H_{241}N_{45}O_{45}$ = 3659.09; calcd. for $[M+2H]^{2+}$ = 1830.54; found =1829.9; calcd. for $[M+3H]^{3+}$ = 1220.7; found = 1220.3; calcd. for $[M+4H]^{4+}$ = 915.7; found = 915.7. calcd. for $[M+5H]^{5+}$ = 732.6; found = 732.7.

UV ($H_2O$) $\lambda_{max}$, nm (ε): 308 (56566) $M^{-1}$ $cm^{-1}$

Yield = 12,07%

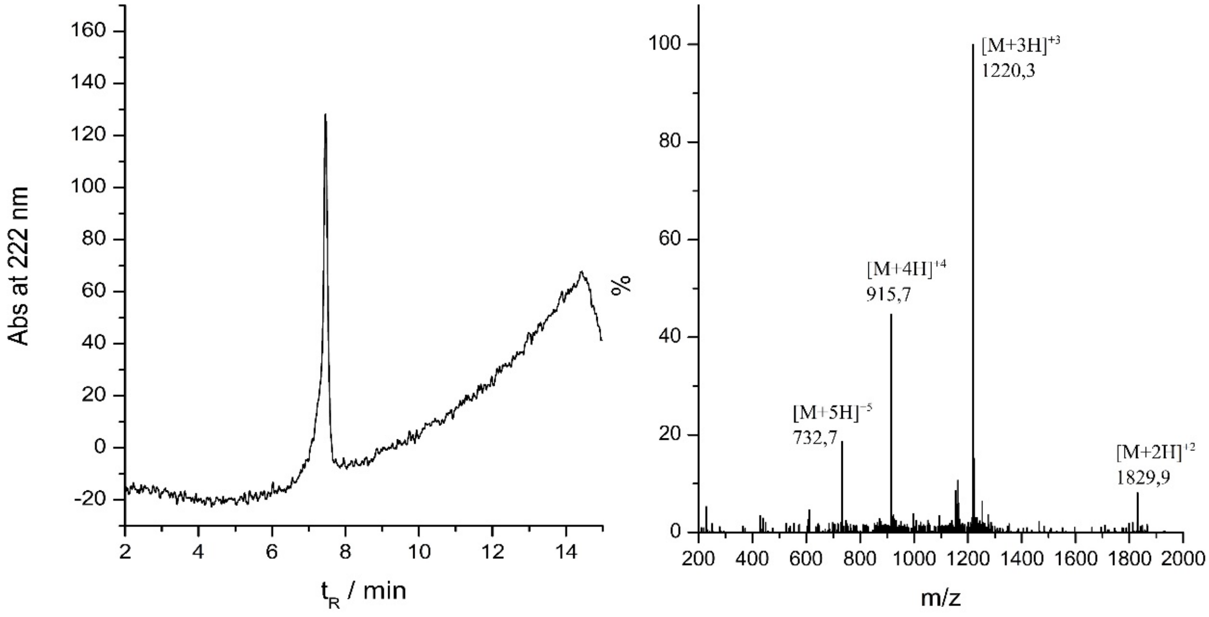


**Figure S1**: Left) HPLC chromatogram of the purified peptide; 5-95% of B in 15 min. Right) Mass spectrum of the purified peptide. Gradient 5 to 75% B over 40 min. (A: $H_2O$ 0.1% TFA, B: $CH_3CN$ 0.1% TFA.

MALDI-TOF (m/z) $[M]^+$ calcd. for $C_{172}H_{241}N_{45}O_{45}$ = 3659.09; found = 3658.90

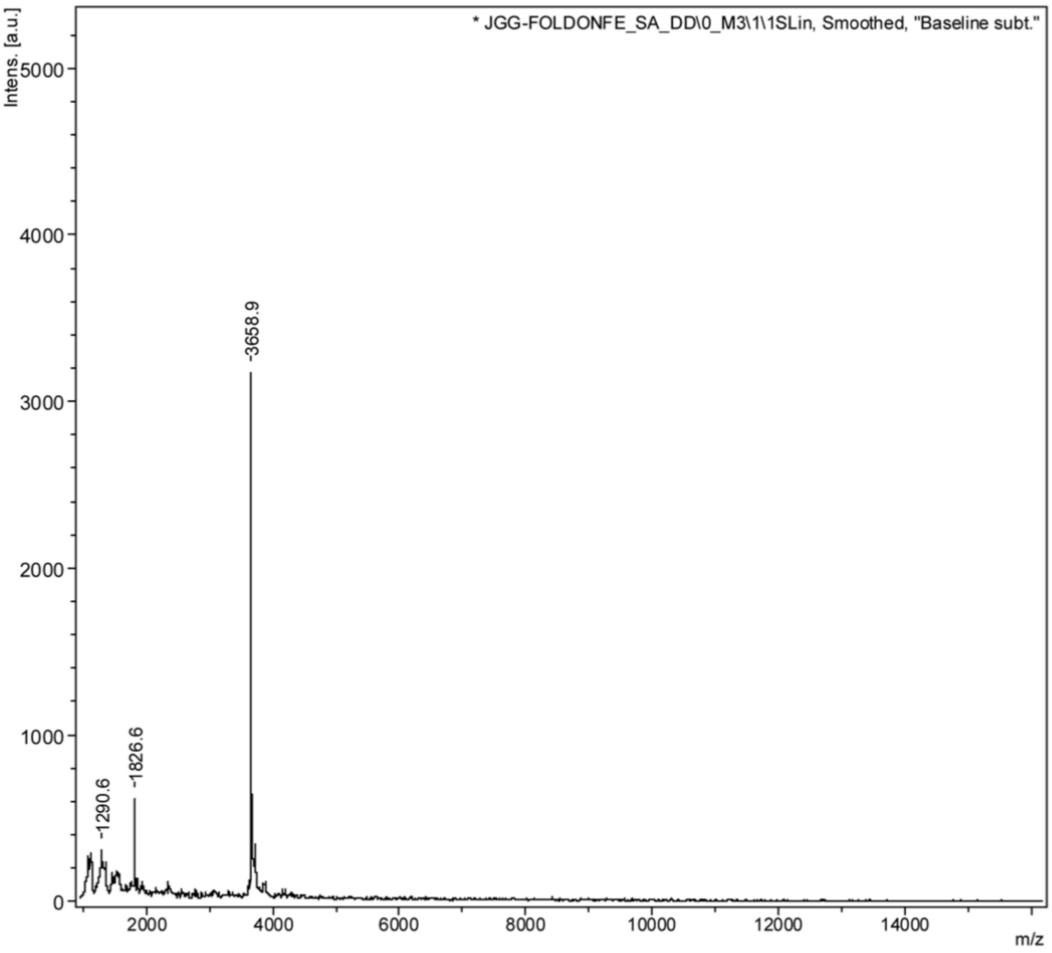


**Figure S2**: MALDI of the purified peptide.

# Helicate synthesis

The peptide helicates were synthetized by addition of aliquots of an aqueous solution containing Fe(II) metal ions (Mohr's salt; the total amount of Fe(II) added was 14 eq.) to a solution of the peptide ligand in 1 mM PBS buffer (pH = 6.5, 293 K).

The helicate peptide was characterized by MALDI-TOF mass spectroscopy:

MALDI-TOF (m/z) $[M]^+$ calcd. for $C_{516}H_{723}N_{135}O_{135}Fe_2$ = 11082.2; found = 11084.60

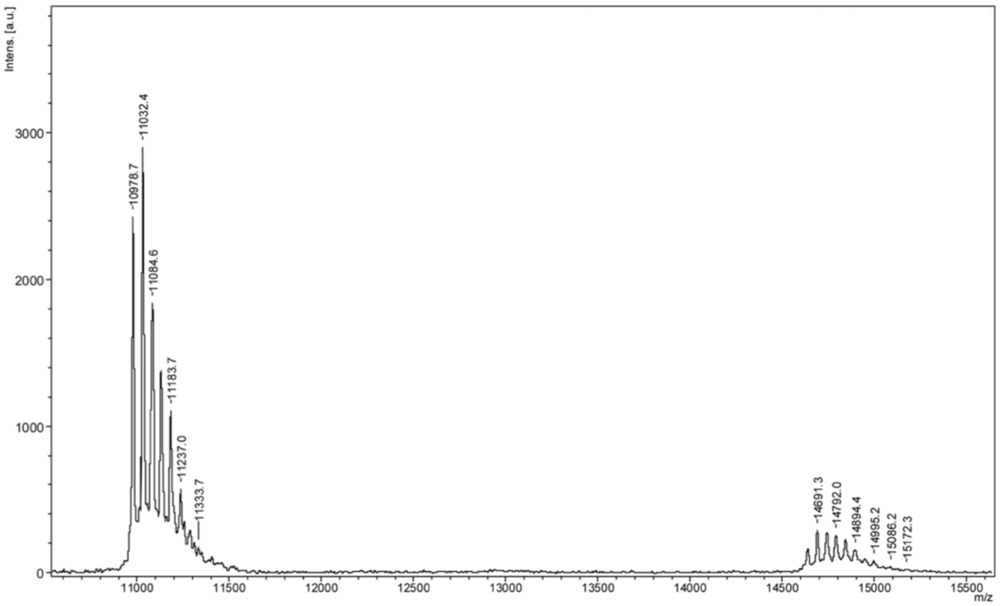


**Figure S3**: MALDI of the trimeric helicate.

**Molecular Dynamics**

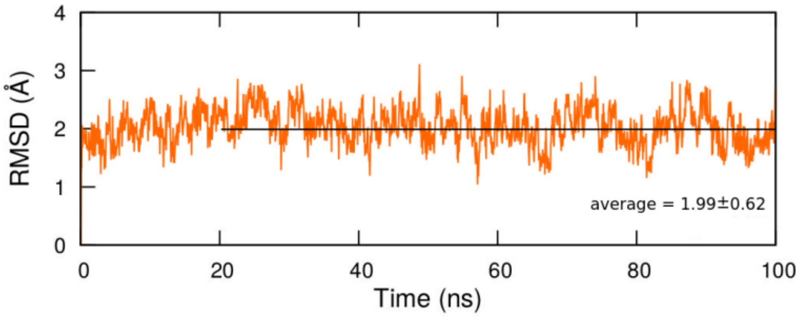


**Figure S4**. Computed RMSD along the MD trajectories for ΛΛ-[(βAlaBpy)$_2$-T4Ff]$_3$Fe$^{+4}$ using the minimized initial structures as a reference. The dispersion in terms of 2σ is also reported.

# Spectrophotometric studies

### Fluorescence emission studies

Over a solution of [**(βAlaBpy)$_2$-T4Ff**]$_3$ (3 μM) in 1 mM PBS buffer, 10 mM NaCl (pH 6.5, 293 K) $(NH_4)_2Fe(SO_4)_2$ • 6 $H_2O$ (Mohr's salt) was added in aliquots until a final concentration of Fe(**II**) = 42 μM (~14 equivalents). In this titration, the quenching of the emission at 420 nm was recorded after the adittion of each aliquot. The data was analysed with *DynaFit* for a 1:2 binding model getting the $K_{D1}$ of 5.5 ± 3.3 μM and a $K_{D2}$ of 6.6 ± 0.7 μM for the coordination of the first and the second Fe(**II**) ion respectively.

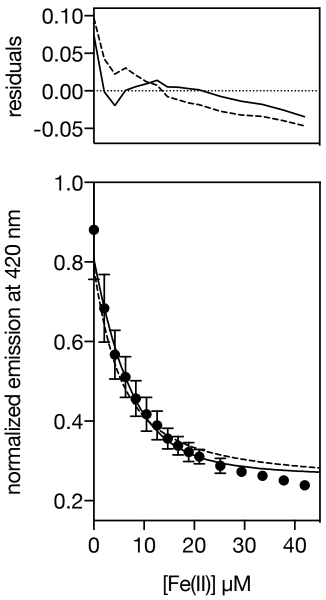


**Figure S5**. Fluorescence titration of a 3 μM (9 μM monomer) solution of [**(βAlaBpy)$_2$-T4Ff**]$_3$ with increasing concentrations of Fe(II) showing the best fit to a 1 to 1 (dashed line) or 1 to 2 (solid line) binding modes. Note the residuals that clearly show a process not captured in the 1 to 1 binding model.

### UV-VIS studies

To confirm the formation of the iron (II) complex, UV-VIS studies were carried out. In these, it was monitored the absorbance at 535 nm before and after the addition of 14 eq of $(NH_4)_2Fe(SO_4)_2 \cdot 6\ H_2O$ to a solution of [**$(\beta$AlaBpy$)_2$-T4Ff**]$_3$ (7 µM) in 1 mM PBS buffer, 10 mM NaCl (pH 6.5, 293 K).

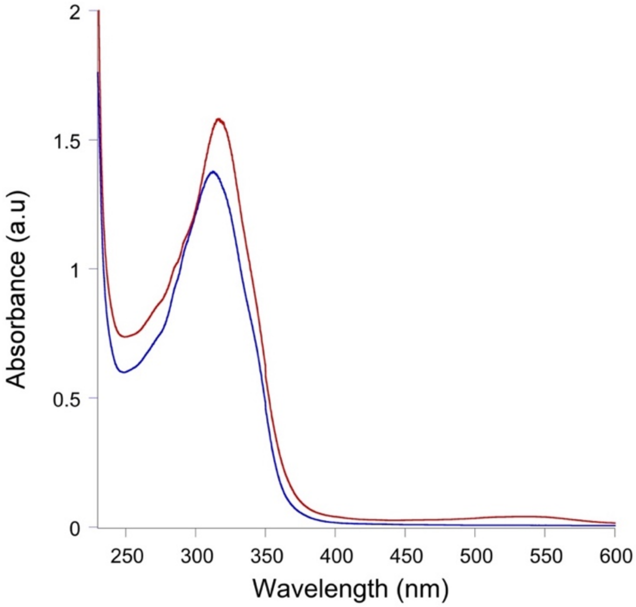


**Figure S6**. UV spectra of [**$(\beta$AlaBpy$)_2$-T4Ff**]$_3$ before (blue line) and after the adition (red line) of 14 eq of Fe(**II**).

### Circular Dichroism

Samples (300 µL, 1 mM PBS buffer, 10 mM NaCl, pH = 6.5, 293 K) contained 6 µM of [**$(\beta$AlaBpy$)_2$-T4Ff**]$_3$ peptides were measured before and after the addition of Fe(**II**) (14 eq, 84 µM in cuvette). The spectra showed are the average of 3 scans.

**Anisotropy titrations with three way junction DNA (twDNA)**

A 2 µM solution of [[**(βAlaBpy)2-T4Ff**]$_3$Fe$_2$]$^{4+}$ in 1 mM PBS buffer (10 mM NaCl, pH 6.5) was titrated with increasing concentrations of the twDNA (final concentration ≈ 20 µM) .The anisotropy signal of the βAlaBpy ($\lambda_{exc}$ = 308, $\lambda_{em}$ = 420 nm) was recorded after each addition, and the resulting profile was analyzed using the program *DynaFit*, considering a 1:1 binding mode and the contribution of non-specific electrostatic complexes to the overall signal ($K_D$ = 2.1 ± 0.61 µM). The experiments were performed at 20 °C in a 1 cm cuvette (excitation slit: 18 nm; emission slit:18 nm; intrgration time: 2s)

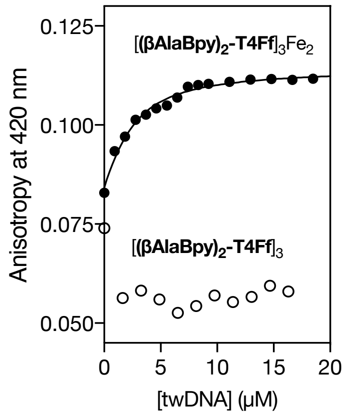


**Figure S7.** Anisotropy titration of 2 µM [[**(βAlaBpy)$_2$-T4Ff**]$_3$Fe$_2$]$^{4+}$ and 2 µM of the foldon [[**(βAlaBpy)$_2$-T4Ff**]$_3$ (in absence of Fe(**II**) ions) in 1 mM phosphate buffer, 10 mM NaCl with increasing concentrations of **tw-DNA**. The best fit to a 1:1 binding mode is shown (curve fitting was performed using *DynaFit*). **twDNA** sequences: 5'–CAC CGC TCT GGT CCT C–3'; 5'–CAG GCT GTG AGC GGT G–3'; 5'–GAG GAC CAA CAG CCT G–3'.

## Anisotropy titrations with ds-DNA

A 2 μM solution of [[**(βAlaBpy)2-T4Ff**]$_3$Fe$_2$]$^{4+}$ in 1 mM PBS buffer (10 mM NaCl, pH 6.5) was titrated with increasing concentrations of the dsDNA (final concentration ≈ 20 μM) .The anisotropy signal of the βAlaBpy ($\lambda_{exc}$ = 308, $\lambda_{em}$ = 420 nm) was recorded after each addition (excitation slit: 18 nm; emission slit:18 nm; intrgration time: 2s).

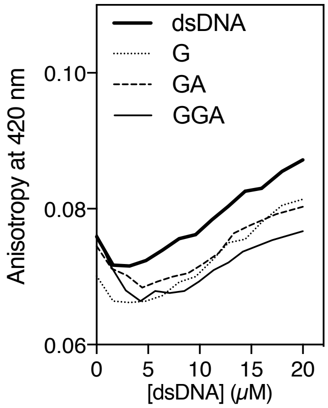


**Figure S8.** Anisotropy titration of 2 μM of the foldon helicate [[**(βAlaBpy)$_2$-T4Ff**]$_3$Fe$_2$]$^{4+}$ in 1 mM phosphate buffer, 10 mM NaCl with increasing concentrations of **dsDNA** and dsDNAs with mistmatched base pairs. The experimental data are represented as lines for clarity. The sequences of the DNAs used were: 5'- AAC ACA TGC AGG ACG GCG CTT-3' ( this is the common strand, that upon annealing with the following sequences gice the standard dsDNA ,as well as the dsDNAs with mismatched G, GA and GGA); **dsDNA** (complementary strand): 5'- AAG CGC CGT CCT GCA TGT GTT-3' (Hebra complementaria a la hebra base); **mismatch G** (complementary strand): 5'-AAG CGC CGT CGT GCA TGT GTT-3'; **mismatch GA** (complementary strand): 5'-AAG CGC CGT CGA GCA TGT GTT-3'; **mismatch GGA** (complementary strand): 5'- AAG CGC CGT GGA GCA TGT GTT-3'.

### DynaFit titration analysis

Experimental data were fitted with the *DynaFit 4.0* software. The program is available free of charge for academia at *http://www.biokin.com/dynafit/* The program requires plain text files called *scripts* that contains information about the chemical model underlying the experimental data, the values of model parameters, such as starting concentrations of reactants, as well as information about location of the files. A typical script used in the analysis titrations is included below. The file has been commented to indicate the purpose of the keywords and sections, but the reader is recommended to review the DynaFit scripting manual distributed along the program or available at the DynaFit website.

```
[task]                              ;semicolons separate comments from actual instructions
 task = fit                         ;nature of the calculation to be performed by DynaFit
 data = equilibria

[mechanism]                         ;We will fit using a simple 1:1 binding model
 R + L <==> RL : Kd dissoc          ;The keyword "dissoc" means it's a dissociation constant
                                    ;The 2:1 mechanism would be indicated as follows:
                                    ;R  + L <==> RL  : Kd1 dissoc
                                    ;RL + L <==> RL2 : Kd2 dissoc

[constants]                         ;Initial Kd value for iteration the question mark
 Kd = 1.0 ?                         ;symbol "?" indicates that the Kd will be optimized
                                    ;in a 2:1 mechanism this section would be:
                                    ;Kd1 = 1.0 ? optimization of Kd1
                                    ;Kd2 = 3.0 ? for optimization of Kd2
[concentrations]
 R = 2.0                            ;Fixed concentration of the receptor in the cuvette.

[responses]                         ;Contribution of each species to the signal.
 intensive                          ;anisotropy values do not depend on the concentration
                                    ;this keyword must be removed when measuring the
                                    ;emission intensity (e.g., Fe(II) coordination).
 R = 0.1 ?
 RL = 1.5 ?                         ;In the 2:1 mechanism we must also include
                                    ;the response of the final RL2 i.e., RL2 = 0.9 ? This
                                    ;can also be linked to RL, (i.e., RL2 = 1.0 * RL)
                                    ;See the DynaFit scripting manual for details.

[data]                              ;Location of files and information about the data
 variable  L                        ;which species changes concentration during titration
 directory ./exp/JGG/tw/data        ;path relative to DynaFit location
 extension txt
 file data_an1 | offset auto ?      ;Name of the experimental data file, and instruction to
                                    ;Automatically offset the data if displaced.
 file data_an2 | offset auto ?      ;Multiple titrations can be fitted simultaneously
                                    ;by adding the files to the list.

[output]
 directory ./exp/JGG/tw/out         ;Path indicating location of out files

[settings]                          ;Cosmetic settings that control DynaFit graphics
 {Output}
 XAxisUnit = uM
 BlackBackground = n
 XAxisLabel = [twDNA, uM]
 YAxisLabel = anisotropy at 420
 WriteTXT = y
```
